# Supplementary material for: Non-Destructive Identification of Dyes on Fabric Using Near-Infrared Raman Spectroscopy
Source: Molecules. 2023 Nov 30;28(23):7864. doi: 10.3390/molecules28237864 (PMC10708237; doi:10.3390/molecules28237864)
Supplement: Supplementary file 1 [file molecules-28-07864-s001.zip › molecules-2735851-supplementary.pdf]

# Non-Destructive Identification of Dyes on Fabric Using Near-Infrared Raman Spectroscopy

Mackenzi Peterson[1] and Dmitry Kurouski[2*]

1. Department of Entomology, Texas A&M University, College Station, Texas 77843, United States
2. Department of Biochemistry and Biophysics, Texas A&M University, College Station, Texas 77843, United States

## Supporting Information

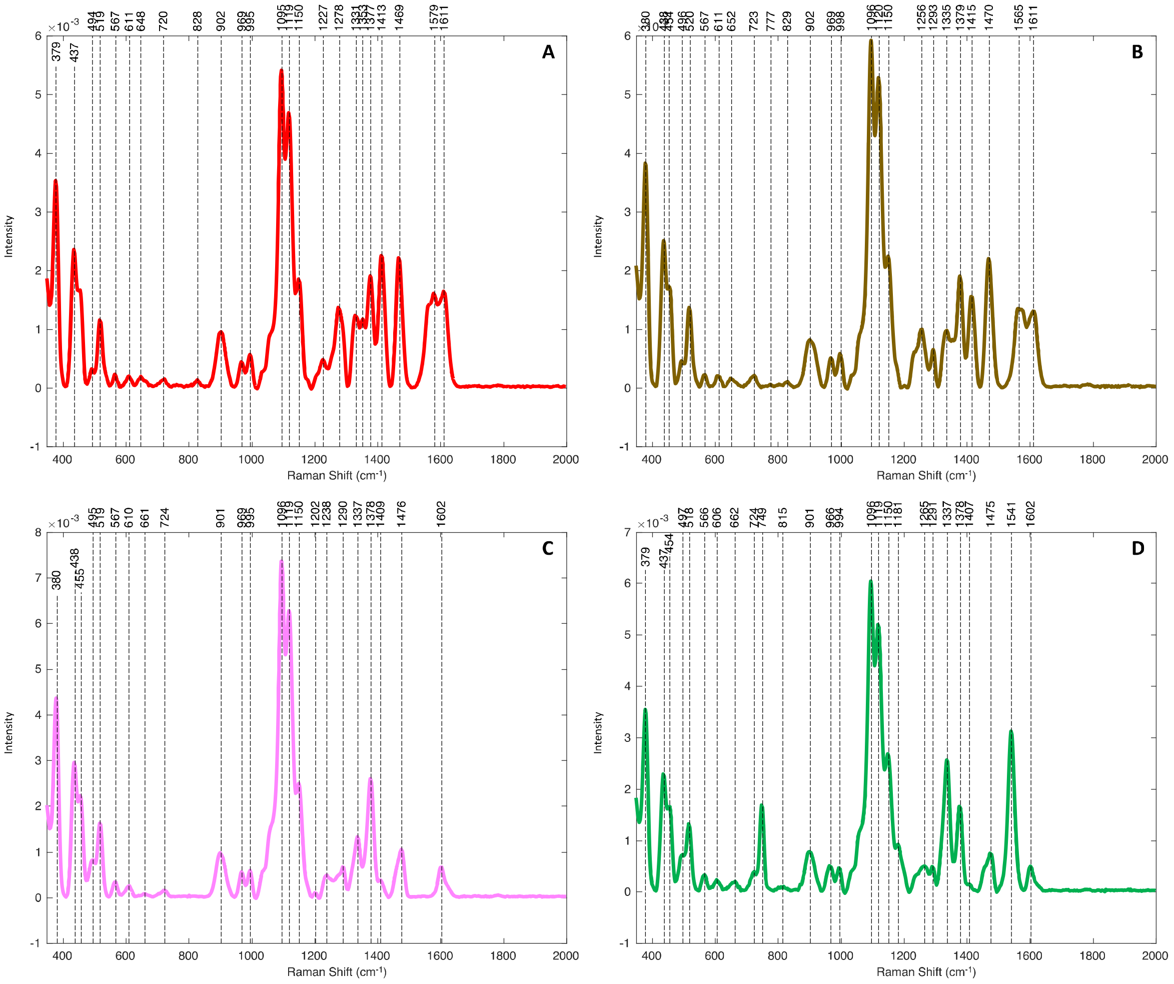

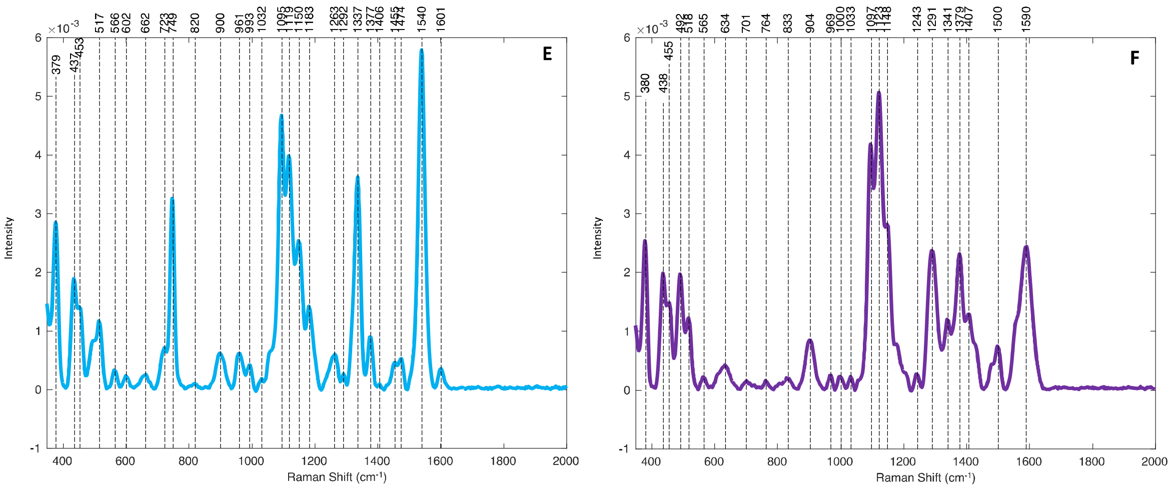


**Figure S1:** Average NIR Spectra of the following dyed cotton samples: A) red B) orange C) yellow D) green E) blue and F) purple

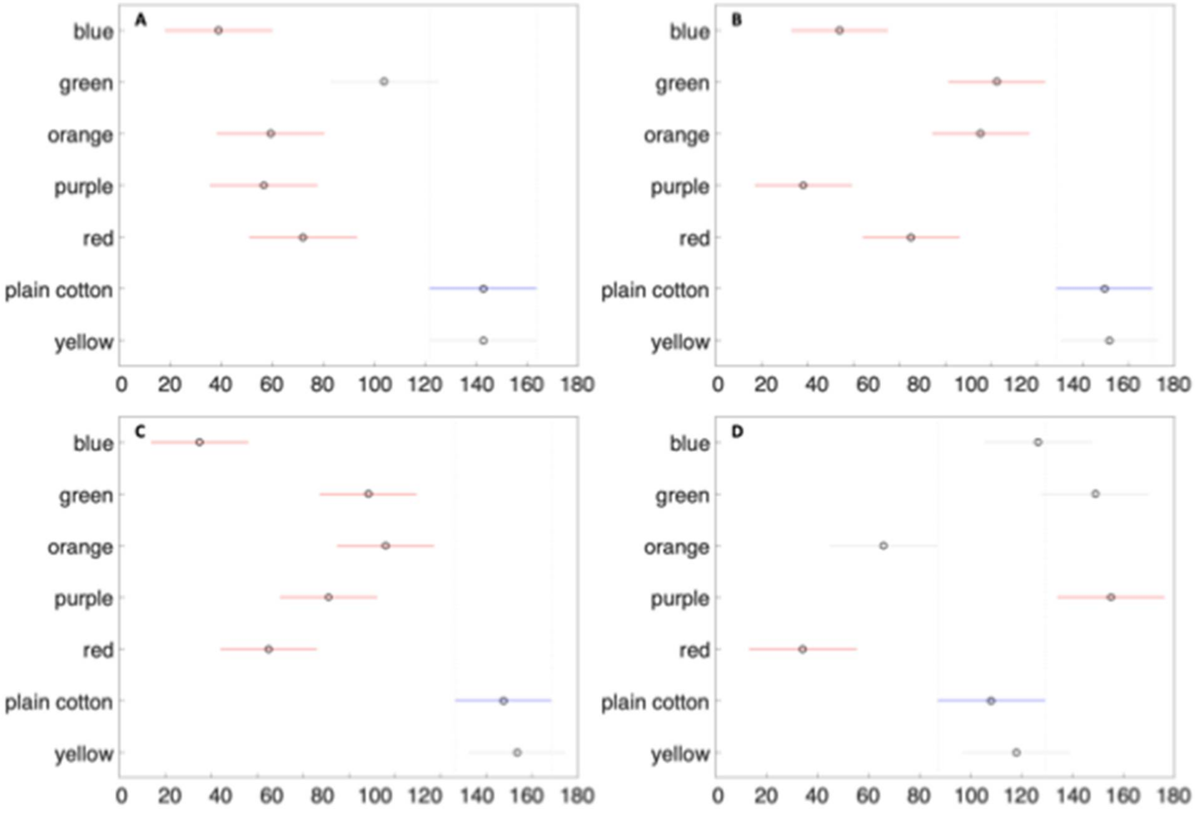


**Figure S2:** ANOVA tests of relative intensities of 1038 $cm^{-1}$ (A) 1096 $cm^{-1}$ (B) 1120 $cm^{-1}$ (C), and 1150 $cm^{-1}$ (D) bands in the Raman spectra acquired from in-lab dyed and undyed cotton fabric.

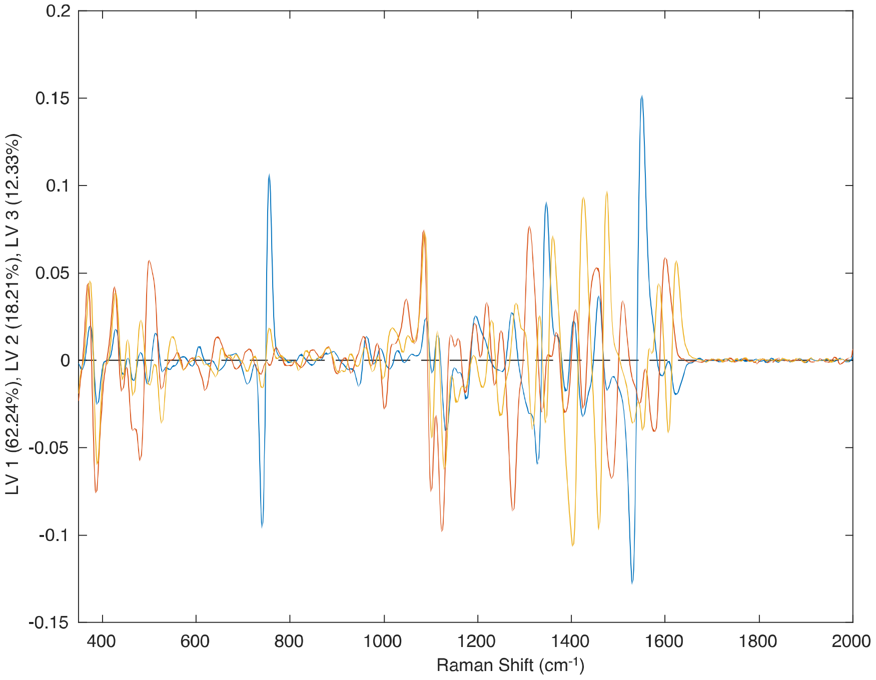


**Figure S3:** LV Plot of In-Lab Dyed Cotton Samples from PLS-DA

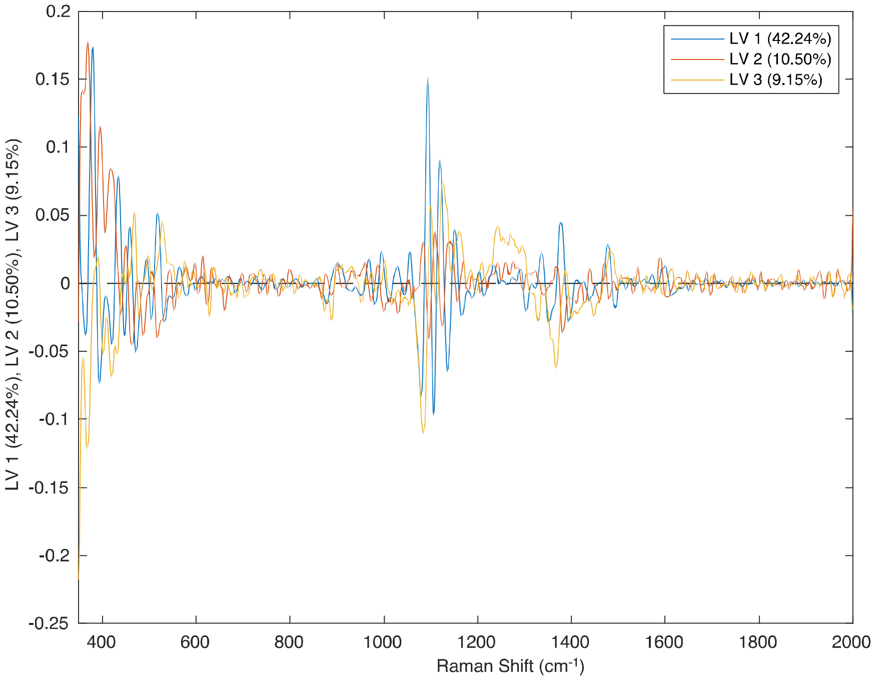


**Figure S4:** LV Plot of Plain Cotton and Yellow In-Lab Dyed Cotton from PLS-DA

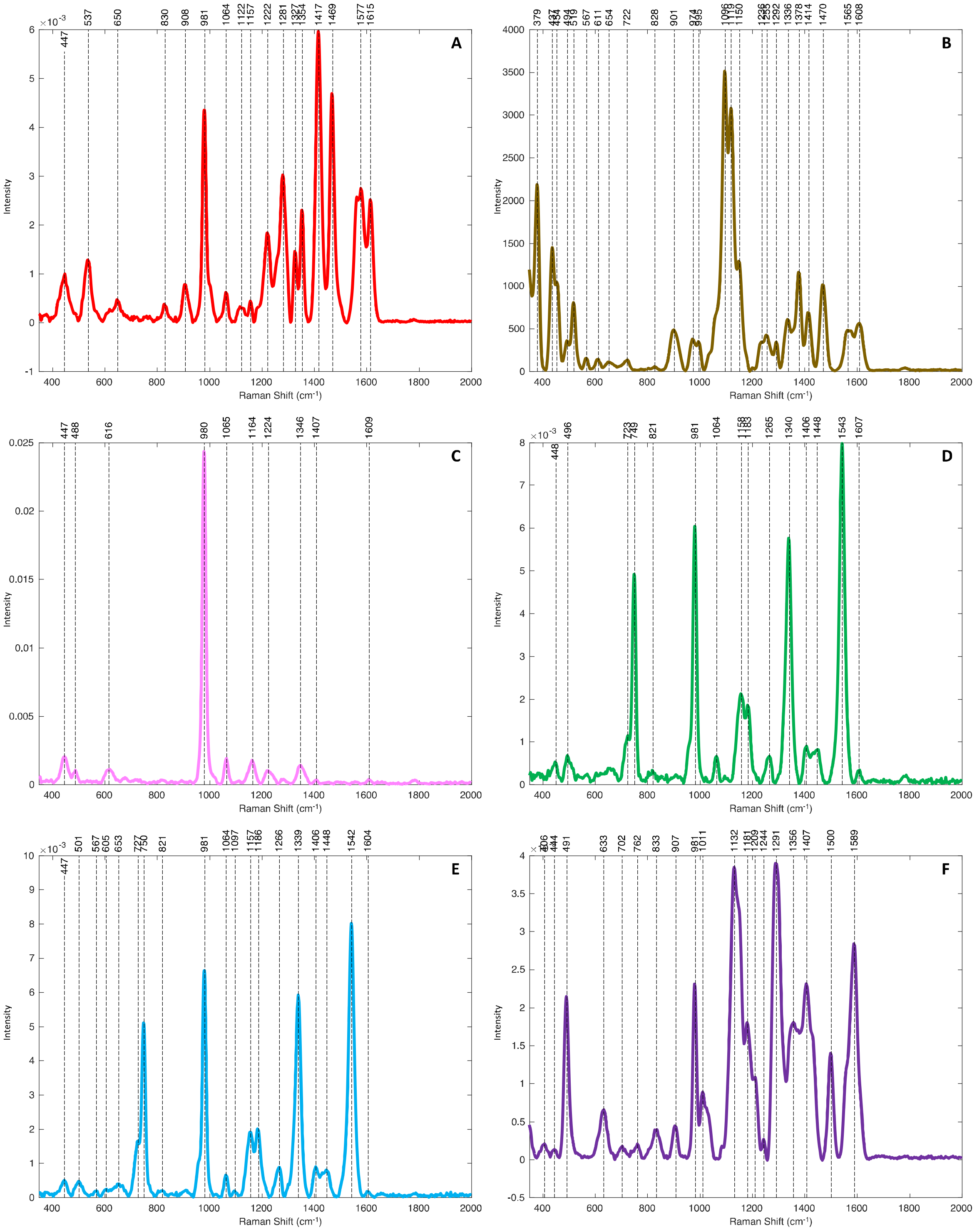


**Figure S5:** Average SORS Spectra of the following liquid dye samples: A) red B) orange C) yellow D) green E) blue and F) purple

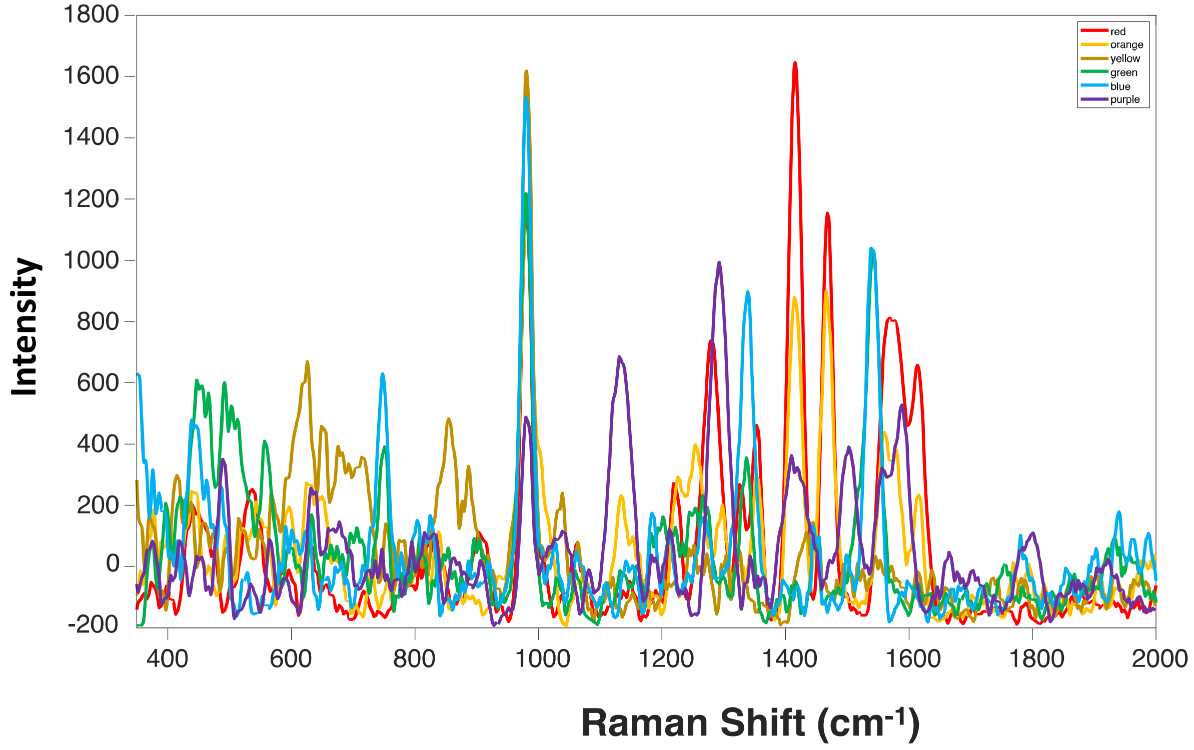


**Figure S6:** NIR Spectra acquired from dyes dissolved in water.

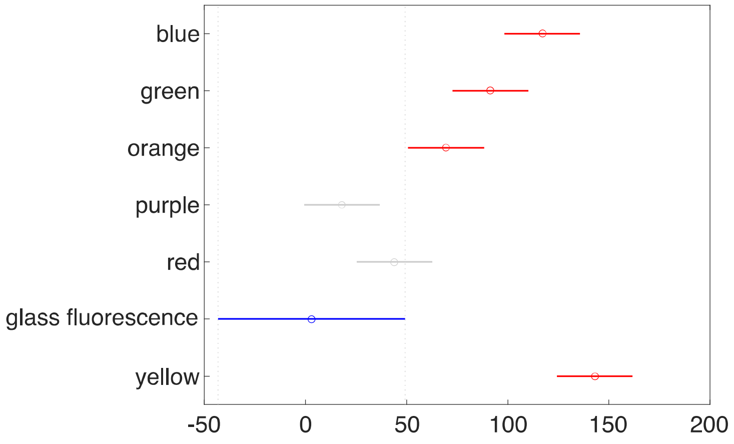


**Figure S7:** ANOVA tests of relative intensities of 980 $cm^{-1}$ in the Raman spectra acquired from dyes dissolved in water.

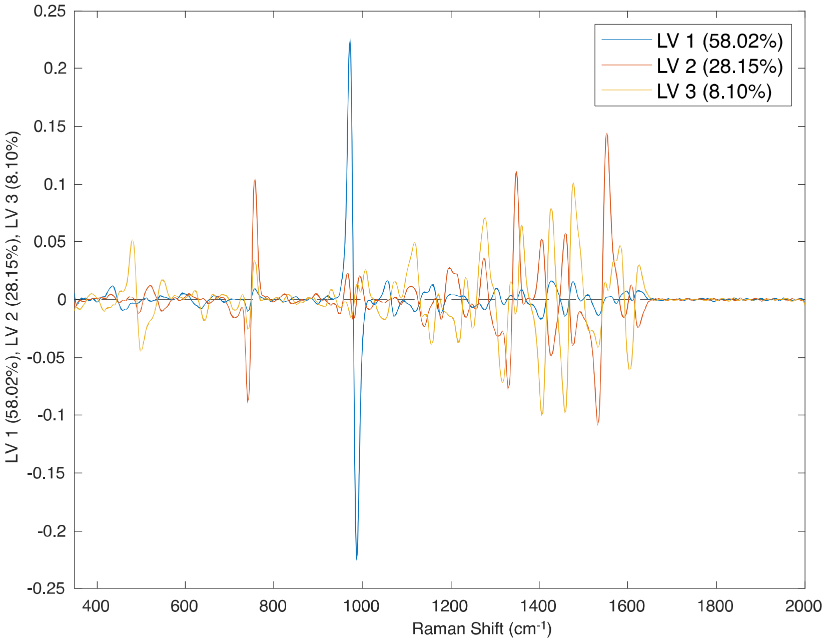


**Figure S8:** LV Plot of Liquid Dye Samples from PLS-DA

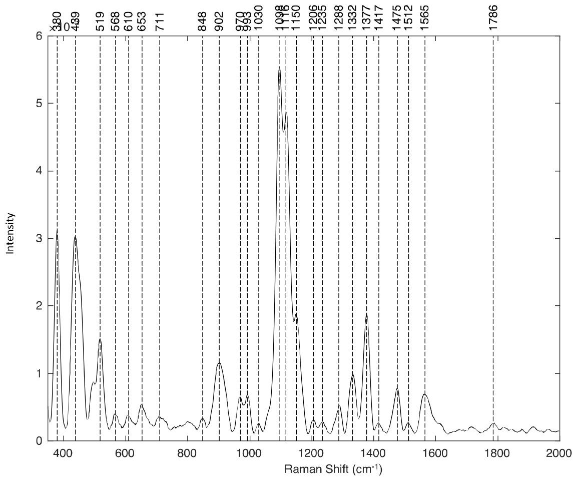


**Figure S9:** Average NIR Spectra of Brazilwood

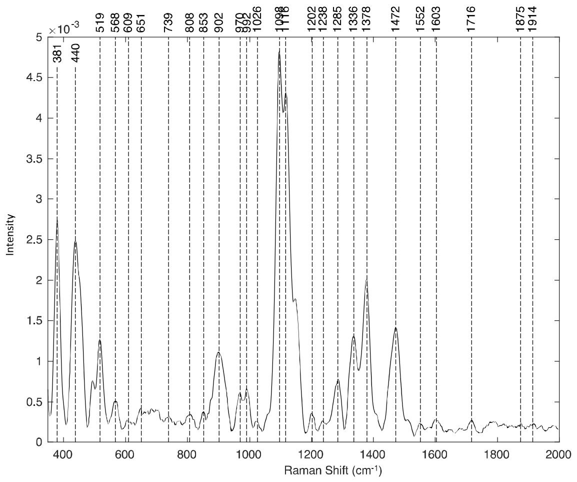


**Figure S10:** Average NIR Spectra of Buckthorn

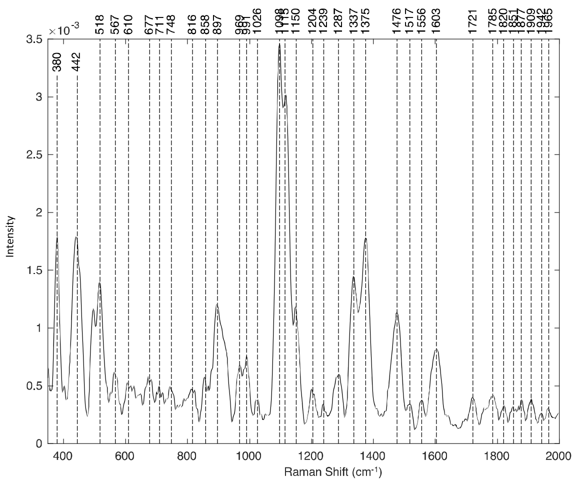


**Figure S11:** Average NIR Spectra of Clutch

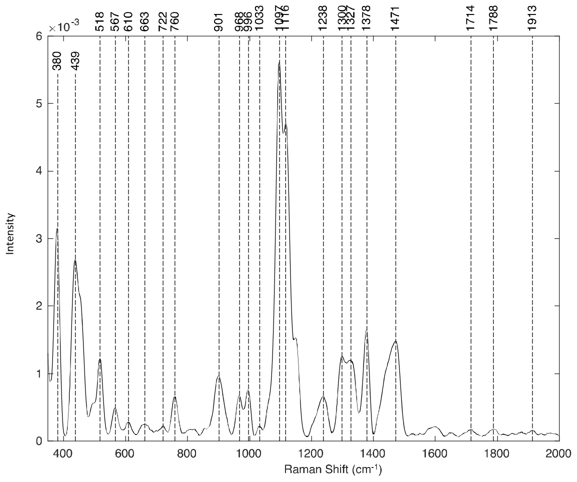

**Figure S12:** Average NIR Spectra of Cochineal

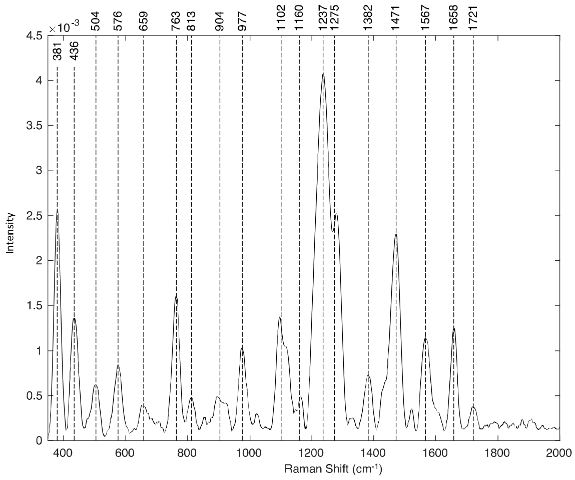


**Figure S13:** Average NIR Spectra of Lac

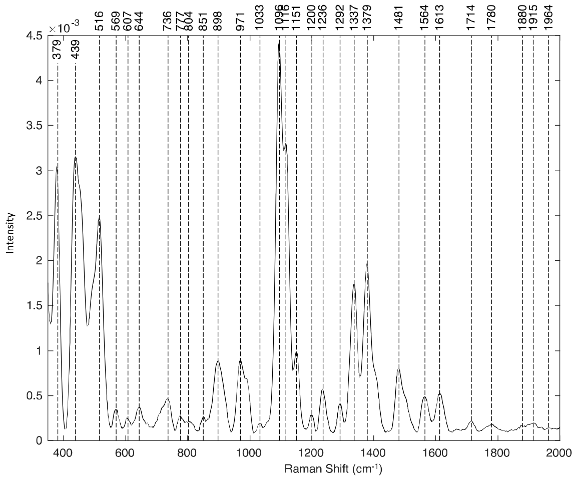


**Figure S14:** Average NIR Spectra of Logwood

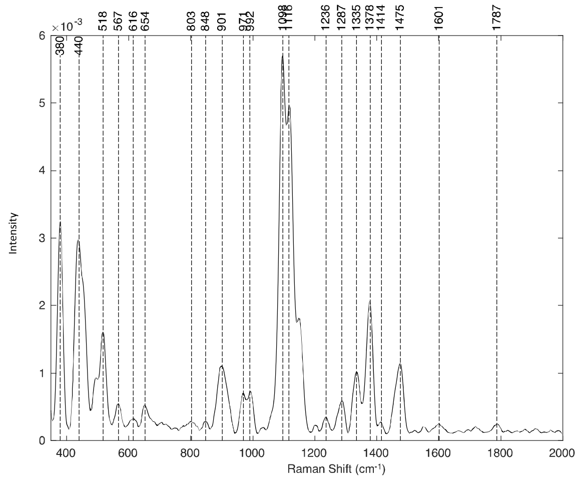


**Figure S15:** Average NIR Spectra of Madder

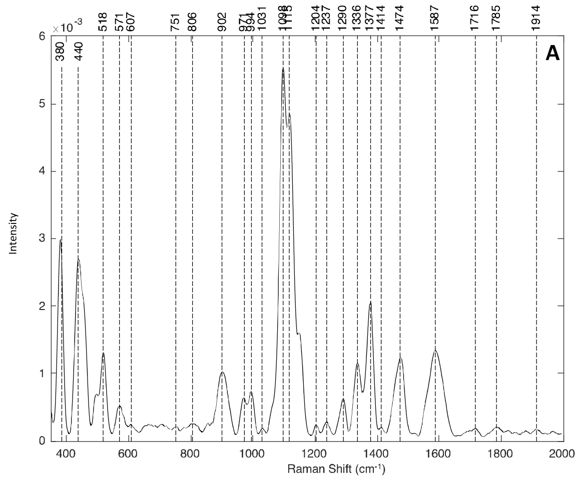


**Figure S16:** Average NIR Spectra of Pomegranate

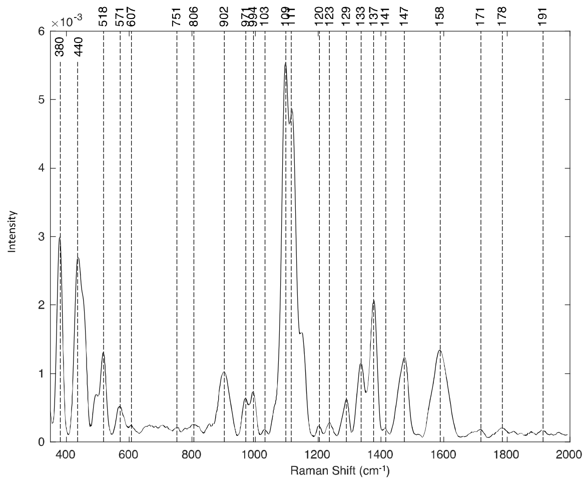

**Figure S17:** Average NIR Spectra of Weld

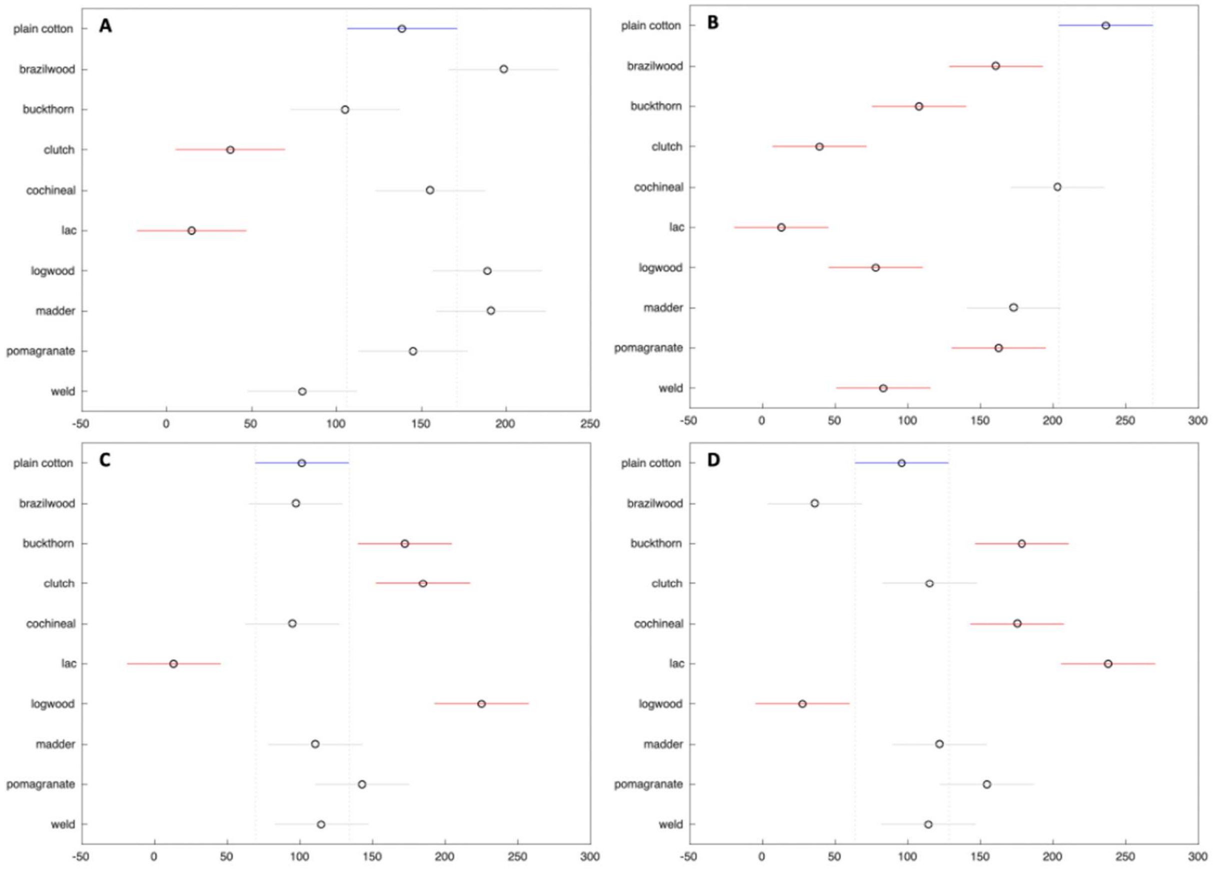


**Figure S18:** ANOVA Tests of Menil Fabric Without Nanoparticles at A) 438 $cm^{-1}$ B) 1097 $cm^{-1}$ C) 1335 $cm^{-1}$ and D)1475 $cm^{-1}$

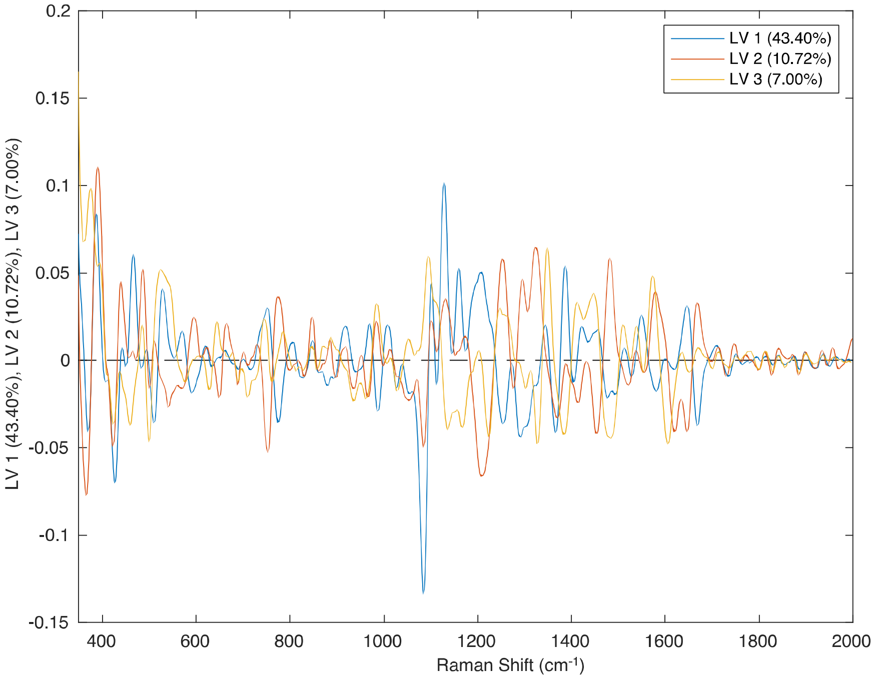


**Figure 19:** LV Plot of Menil Fabric Without Nanoparticles From PLS-DA
